# Supplementary material for: The Reversible Methylation of m6A Is Involved in Plant Virus Infection
Source: Biology (Basel). 2022 Feb 9;11(2):271. doi: 10.3390/biology11020271 (PMC8869485; doi:10.3390/biology11020271)
Supplement: Supplementary file 1 [file biology-11-00271-s001.zip › biology-1512574-supplementary.pdf]

Supplementary Table S1 Methylation-related proteins and functions

**Name**	**Species**	**Function**	**References**
METTL3	mammalian	S-adenosyl methionine binding subunit. Its deletion can causes early embryonic lethality in mice.	[1]
METTL14	mammalian	A component of the m6A methyltransferase complex. The heterodimer METTL3-METTL14 shows much higher methyltransferase activity.	[2]
METTL16	human	A U6 spliceosomal small nuclear RNA (snRNA) A43-N6 -methyltransferase. It can methylate mRNA as well as U6 snRNA and various lncRNAs in humans.	[3] [4]
WTAP	human	Wilms' tumor 1-associating protein was regarded as a binding protein of Wilms' tumor 1 affecting splicing. It plays a important role in m6A deposition.	[5]
Fl(2)d	*Drosophila melanogaster*	The homolog of WTAP. Affects pre-mRNA splicing and highly expressed in reproductive organs.	[6]
KIAA1429/VIRMA	*Drosophila melanogaster*	It was shown to mediates methylation in the 3'UTR and around the stop codon to affecting alternative polyadenylation and involved in m6A formation in mammals.	[7] [8]
Ime4	*Saccharomyces cerevisiae*; *Drosophila melanogaster*	METTL3 homolog. A single amino acid mutant in the catalytic residues of Ime4 can cause sporulation defects in *Saccharomyces Cerevisiae* and controls sex determination in *Drosophila melanogaster*.	[9, 10]
MTA	*Arabidopsis thaliana*	METTL3 homolog, T-DNA insertion mutant of MTA shows an embryo lethal phenotype.	[11, 12]
MTB	*Arabidopsis thaliana*	It may display enzymatic activity in *Arabidopsis*. m6A levels reduced in an inducible MTB RNAi line.	[13, 14]
HAKAI	*Arabidopsis thaliana* and human	E3-ubiquitin ligase, it can interact with WTAP, In *Arabidopsis*, HAKAI can be co-immunoprecipitated by FIP37 and m6A levels reduced in HAKAI mutant lines. Knockdown of HAKAI in human cell decreases the m6A of polyA-RNA.	[7, 13]
RBM15	mammalian	Interacts with WTAP and involved in m6A methylation. Deletion of RBM15 is embryo lethal in mice. Knockdown of RBM15 and its paralog RBM15B decreases	[15, 16]

		m6A level and impairs XIST-mediated X-inactivation.	
FPA	*Arabidopsis thaliana*	The homolog of RBM15. Regulates flowering time by RNA-mediated chromatin silencing of the floral repressor FLC.	[17, 18]
FIP37	*Arabidopsis thaliana*	Interacts with MTA and is essential to mediate m6A mRNA modification of key shoot meristem genes	[11, 19]
FTO	mammalian	The first m6A demethylase and the known body mass index and obesity-associated gene. In human, loss of FTO can causes growth retardation and deformities in human.	[20, 21]
ALKBH5	mammalian	ALKBH5 belongs to the Fe(II)/α-KG-dependent dioxygenase superfamily. It affects nascent mRNA synthesis and the splicing rate in human cells.	[22]
atALKBH9B	*Arabidopsis thaliana*	m6A RNA demethylases and it's activity affects the m6A level in the AMV genome.	[23]
atALKBH10B	*Arabidopsis thaliana*	Affects m6A levels in endogenous polyA-RNA. atALKBH10B mutant delays flowering and represses vegetative growth.	[24]

References:

1. Geula S.; Moshitchmoshkovitz S.; Dan D.; Mansour A.F.; Kol N.; Salmondivon M.; Hershkovitz V.; Peer E.; Mor N.; Manor Y.S. m6A mRNA methylation facilitates resolution of naive pluripotency toward differentiation. *Science*. **2015**, 347, 1002-1006.
2. Liu J.; Yue Y.; Han D.; Wang X.; Fu Y.; Zhang L.; Jia G.; Yu M.; Lu Z.; Deng X.; Dai Q.; Chen W.; He C. A METTL3-METTL14 complex mediates mammalian nuclear RNA N6-adenosine methylation. *Nat Chem Biol*. **2014**, 10, 93-95.
3. Warda A.S.; Kretschmer J.; Hackert P.; Lenz C.; Urlaub H.; Höbartner C.; Sloan K.E.; Bohnsack M.T. Human METTL16 is a N(6)-methyladenosine (m(6)A) methyltransferase that targets pre-mRNAs and various non-coding RNAs. *Embo Rep.* **2017**, 18, 2004-2014.
4. Pendleton K.E.; Chen B.; Liu K.; Hunter O.V.; Xie Y.; Tu B.P.; Conrad N.K. The U6 snRNA m(6)A Methyltransferase METTL16 Regulates SAM Synthetase Intron Retention. *Cell*. **2017**, 169, 824-835.
5. Little N.A.; Hastie N.D.; Davies R.C. Identification of WTAP, a novel Wilms' tumour 1-associating protein. *Hum. Mol. Genet.* **2000**, 9, 2231-2239.
6. Granadino B.; Campuzano S.; Sánchez L. The Drosophila melanogaster fl(2)d gene is needed for the female-specific splicing of Sex-lethal RNA. *Embo J.* **1990**, 9, 2597-2602.

7. Yue Y.; Liu J.; Cui X.; Cao J.; Luo G.; Zhang Z.; Cheng T.; Gao M.; Shu X.; Ma H.; Wang F.; Wang X.; Shen B.; Wang Y.; Feng X.; He C.; Liu J. VIRMA mediates preferential m(6)A mRNA methylation in 3'UTR and near stop codon and associates with alternative polyadenylation. *Cell Discov*. **2018**, 4, 10.
8. Schwartz S.; Mumbach M.R.; Jovanovic M.; Wang T.; Maciag K.; Bushkin G.G.; Mertins P.; Ter-Ovanesyan D.; Habib N.; Cacchiarelli D.; Sanjana N.E.; Freinkman E.; Pacold M.E.; Satija R.; Mikkelsen T.S.; Hacohen N.; Zhang F.; Carr S.A.; Lander E.S.; Regev A. Perturbation of m6A writers reveals two distinct classes of mRNA methylation at internal and 5' sites. *Cell Rep*. **2014**, 8, 284-296.
9. Hongay C.F.; Orr-Weaver T.L. Drosophila Inducer of MEiosis 4 (IME4) is required for Notch signaling during oogenesis. *P. Natl Acad. Sci. Usa*. **2011**, 108
10. Haussmann I.U.; Bodi Z.; Sanchez-Moran E.; Mongan N.P.; Archer N.; Fray R.G.; Soller M. m(6)A potentiates Sxl alternative pre-mRNA splicing for robust Drosophila sex determination. *Nature*. **2016**, 540, 301-304.
11. Zhong S.; Li H.; Bodi Z.; Button J.; Vespa L.; Herzog M.; Fray R.G. MTA is an Arabidopsis messenger RNA adenosine methylase and interacts with a homolog of a sex-specific splicing factor. *Plant Cell*. **2008**, 20, 1278-1288.
12. Bodi Z.; Zhong S.; Mehra S.; Song J.; Graham N.; Li H.; May S.; Fray R.G. Adenosine Methylation in Arabidopsis mRNA is Associated with the 3'End and Reduced Levels Cause Developmental Defects. *Front Plant*. **2012**, 3, 48.
13. Růžička K.; Zhang M.; Campilho A.; Bodi Z.; Kashif M.; Saleh M.; Eeckhout D.; El-Showk S.; Li H.; Zhong S.; De Jaeger G.; Mongan N.P.; Hejátko J.; Helariutta Y.; Fray R.G. Identification of factors required for m(6) A mRNA methylation in Arabidopsis reveals a role for the conserved E3 ubiquitin ligase HAKAI. *New Phytol.* **2017**, 215, 157-172.
14. Balacco D.L.; Soller M. The m(6)A Writer: Rise of a Machine for Growing Tasks. *Biochemistry-Us*. **2019**, 58, 363-378.
15. Raffel G.D.; Mercher T.; Shigematsu H.; Williams I.R.; Cullen D.E.; Akashi K.; Bernard O.A.; Gilliland D.G. Ott1(Rbm15) has pleiotropic roles in hematopoietic development. *Proc Natl Acad Sci U S A*. **2007**, 104, 6001-6006.
16. Patil D.P.; Chen C.K.; Pickering B.F.; Chow A.; Jackson C.; Guttman M.; Jaffrey S.R. m(6)A RNA methylation promotes XIST-mediated transcriptional repression. *Nature*. **2016**, 537, 369-373.
17. Baeurle I.; Smith L.; Baulcombe D.C.; Dean C. Widespread Role for the Flowering-Time Regulators FCA and FPA in RNA-Mediated Chromatin Silencing. *Science*. **2007**, 318, 109-112.
18. Hornyik C.; Duc C.; Rataj K.; Terzi L.C.; Simpson G.G. Alternative polyadenylation of antisense RNAs and flowering time control. *Biochem Soc Trans*. **2010**, 38, 1077-1081.
19. Shen L.; Liang Z.; Gu X.; Chen Y.; Teo Z.W.; Hou X.; Cai W.M.; Dedon P.C.; Liu L.; Yu H. N6-Methyladenosine RNA Modification Regulates Shoot Stem Cell Fate in Arabidopsis. *Dev. Cell*. **2016**,
20. Dina C.; Meyre D.; Gallina S.; Durand E.; Körner A.; Jacobson P.; Carlsson L.M.; Kiess W.; Vatin V.; Lecoeur C.; Delplanque J.; Vaillant E.; Pattou F.; Ruiz J.; Weill J.; Levy-Marchal C.; Horber F.; Potoczna N.; Hercberg S.; Le Stunff C.; Bougnères P.; Kovacs P.; Marre M.; Balkau B.; Cauchi S.; Chèvre J.C.; Froguel P. Variation in FTO contributes to childhood obesity and severe adult obesity. *Nat. Genet.* **2007**, 39, 724-726.

21. Boissel S.; Reish O.; Proulx K.; Kawagoe-Takaki H.; Sedgwick B.; Yeo G.S.; Meyre D.; Golzio C.; Molinari F.; Kadhom N.; Etchevers H.C.; Saudek V.; Farooqi I.S.; Froguel P.; Lindahl T.; O'Rahilly S.; Munnich A.; Colleaux L. Loss-of-function mutation in the dioxygenase-encoding FTO gene causes severe growth retardation and multiple malformations. *Am. J. Hum. Genet.* **2009**, 85, 106-111.
22. Zheng G.; Dahl J.A.; Niu Y.; Fedorcsak P.; Huang C.M.; Li C.J.; Vågbø C.B.; Shi Y.; Wang W.L.; Song S.H.; Lu Z.; Bosmans R.P.; Dai Q.; Hao Y.J.; Yang X.; Zhao W.M.; Tong W.M.; Wang X.J.; Bogdan F.; Furu K.; Fu Y.; Jia G.; Zhao X.; Liu J.; Krokan H.E.; Klungland A.; Yang Y.G.; He C. ALKBH5 is a mammalian RNA demethylase that impacts RNA metabolism and mouse fertility. *Mol. Cell*. **2013**, 49, 18-29.
23. Martínez-Pérez M.; Aparicio F.; López-Gresa M.P.; Bellés J.M.; Sánchez-Navarro J.A.; Pallás V. Arabidopsis m(6)A demethylase activity modulates viral infection of a plant virus and the m(6)A abundance in its genomic RNAs. *Proc Natl Acad Sci U S A*. **2017**, 114, 10755-10760.
24. Duan H.C.; Wei L.H.; Zhang C.; Wang Y.; Chen L.; Lu Z.; Chen P.R.; He C.; Jia G. ALKBH10B is An RNA N6-Methyladenosine Demethylase Affecting Arabidopsis Floral Transition. *The Plant Cell*. **2017**, 912-2016.
